# Fully automated in vivo screening system for multi-organ imaging and pharmaceutical evaluation

**DOI:** 10.1038/s41378-024-00852-9

**Published:** 2025-01-27

**Authors:** Junhan Duan, Guanming Lin, Kangjian Jiao, Xiaohui Hong, Xudong Lin

**Affiliations:** Guangdong Provincial Key Laboratory of Sensor Technology and Biomedical Instrument, School of Biomedical Engineering, Shenzhen Campus of Sun Yat-Sen University, 518000 Shenzhen, China

**Keywords:** Engineering, Chemistry

## Abstract

Advancements in screening technologies employing small organisms have enabled deep profiling of compounds in vivo. However, current strategies for phenotyping of behaving animals, such as zebrafish, typically involve tedious manipulations. Here, we develop and validate a fully automated in vivo screening system (AISS) that integrates microfluidic technology and computer-vision-based control methods to enable rapid evaluation of biological responses of non-anesthetized zebrafish to molecular gradients. Via precise fluidic control, the AISS allows automatic loading, encapsulation, transportation and immobilization of single-larva in droplets for multi-organ imaging and chemical gradients generation inaccessible in previous systems. Using this platform, we examine the cardiac sensitivity of an antipsychotic drug with multiple concentration gradients, and reveal dramatic diversity and complexity in the accurate chemical regulation of cardiac functions in vivo. This proposed system expands the arsenal of tools available for in vivo screening and facilitates comprehensive profiling of pharmaceuticals.

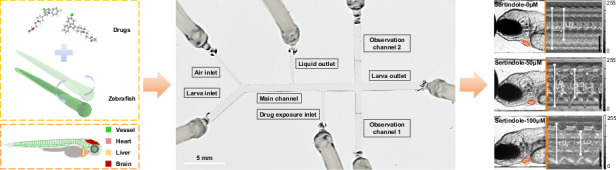

## Introduction

Preclinical evaluation of drug-induced cardiotoxicity holds significant importance in drug development^1–4^. Current strategies for screening candidate drugs for identifying the cardiac toxicity effects focus on cardiomyocyte-based in vitro assays^5–7^. While most of technologies permit rapid screening, they fail to reflect the impact on the organ systems in animals. Recent studies have shown possibilities to deeply evaluate the cardiac toxicity of compounds using the small organisms^8–10^.

Owing to the small size, optically transparent organ systems, high degree of conservation to mammals and rapid development process, zebrafish (Danio rerio) serves as an ideal organism model for high-throughput in vivo evaluation of chemicals that cannot be replicated in vitro^11–16^. Importantly, the blood circulation of zebrafish embryos begins only one day post-fertilization (dpf)^17^. And its ventricle-to-atrial ratio and heart rates are also comparable to those of mammals, respectively^18,19^. These features make zebrafish an important tool for assessing drug cardiotoxicity in vivo^20,21^. However, traditional profiling of cardiac functions in zebrafish still largely relies on time-consuming manipulation and anesthetic treatments to achieve proper orientation and immobilization, which is inefficient and unreliable, particularly unsuitable for large-scale screening. Additionally, the manual operations induced interferences, to a certain extent, limit their practical applications in drug discovery.

Recent advancements in microfluidics and automatic control technology have realized automated drug screening systems using zebrafish larvae. Pardo-Martin et al. developed a system capable of imaging zebrafish larva in any orientation within a glass tube at cellular resolution^22^. Although this platform is powerful, it requires anesthetic treatments in animals, which can decrease heart rate^23^. Additionally, the complex and costly control system hinders its applications in drug screening, especially when targeting specific organ systems like the heart. Chen et al. proposed a non-contact method using acoustofluidic rotational tweezers to manipulate and observe zebrafish larvae^24^. Nevertheless, this system also requires anesthesia for the larvae. Candelier et al. enabled the semi-immobilization of individual zebrafish using microfluidic technology for on-chip delivering and evaluating multiple chemical stimuli^25,26^. However, the manual agarose-based strategies result in low efficiency in such platform. To enable high-throughput screening using zebrafish larvae, we have proposed a fish-trapping technology to immobilize multiple larvae in a microfluidic array with specific orientation based on hydrodynamic forces recently^27^. But the continuous fluidics induced large drug consumption and no orthogonal dimension for generating chemical gradients also limit its practical utility for drug evaluations with real-time recording of cardiac activity in vivo.

Here, we present the design of an automated in vivo screening system (AISS) for fully automatic loading, transportation, orientation and immobilization of zebrafish larvae. It combines microfluidic systems and automated control technologies to achieve animal manipulation, chemical delivering and optical evaluations. A fully automated computer-vision based screening scheme with stable and precise microfluidic control is demonstrated. Specifically, single-larva is encapsulated into a droplet in the microfluidic system facilitating rapid drug perfusion with concentration gradients. Moreover, coupling with the specific design in observation channels in the AISS, immobilization of each single-larva with specific orientation is achieved without any agarose gel fixation or anesthesia treatments, allowing real-time visualization of any organ system based on multispectral microscopic imaging. To validate the functionality of the AISS, we examine cardiac sensitivity to sertindole, an atypical antipsychotic, with multiple concentration gradients and reveal dramatic diversity and complexity in the accurate chemical regulation of cardiac functions. Notably, to our best knowledge, it is the first time to dissect the cardiac sensitivity of sertindole in such in vivo assay. We envision our AISS will significantly expand the toolkit for automated in vivo screening, thereby broadening the applications of the zebrafish model across a wide range of biomedical and biological fields.

## Results

### System design of AISS

**Fig. 1:**
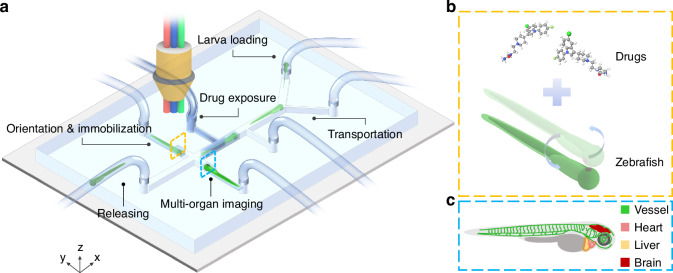
The design of the AISS. **a**–**c** Schematic illustrations of the automated in vivo screening system (AISS) for rapid loading, trapping, orientation of zebrafish larvae (**a**). The precise manipulation of larvae enabled animal immobilization with controllable multi-concentration gradient drug exposure (**b**) and multi-organ imaging (**c**) within the microfluidic chip

To achieve the fully automatic loading, transportation, drug perfusion, and optical evaluation of zebrafish larvae, we developed an automated in vivo screening system (AISS), combining microfluidic technology with computer vision-based closed-loop control strategies (Fig. 1). By carefully controlling the pumps and valves, the AISS allowed precise and rapid generation of droplets, which was utilized for zebrafish immobilization and drug perfusion in the microfluidic system, and further enabled multi-organ imaging and functional screening. Differing from previous methods that employed rigid glass tubes or agarose gel for zebrafish immobilization^22,25,26,28^, the AISS trapped single-animal in droplets and orientated the larvae within the microfluidic channels without any anesthetic treatment, which was important for functional evaluations of the chemoresponsive organ systems including the heart, vessel and brain.

**Fig. 2:**
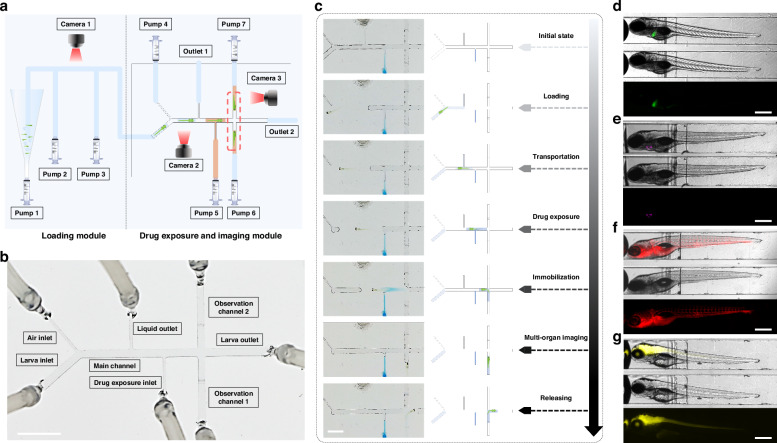
The design of the microfluidic system in the AISS. **a** Schematic of the AISS, including loading module and drug exposure and imaging module. **b** Photograph of the microfluidic chip connected with fluidic capillaries. Scale bar, 5 mm. **c** Photographs and corresponding schematic diagrams showing automated manipulation of a single larva in the AISS. Scale bar, 3 mm. **d**–**g** Multi-organ imaging enabled by the AISS. Representative merged/white-field/ fluorescent images of liver, heart, vessel and brain in the Apo14: GFP (**d**), Cmlc2:eGFP (**e**), Kdrl1:eGFP (**f**), and HuC:eGFP (**g**) transgenic animals, respectively. Scale bar, 500 µm

As shown in Fig. 2a, the AISS was developed based on a motorized X-Y-Z stage with Light Emitting Diode (LED) light sources interconnected via silicone tubing. The microfluidic pumps, driven by stepper motors, were linked to electric microfluidic valves at the end of each tube. And three Complementary Metal-Oxide-Semiconductor (CMOS) cameras were employed in the AISS to enable computer vision-based closed-loop control. In the loading module, zebrafish larvae are loaded from a tapered reservoir with an air bubble generator, which was used to lift randomly distributed zebrafish into the negative-pressure silicone tubing by the rising bubbles from the bottom of the reservoir (Supplementary Fig. 1). Camera 1 was strategically placed between Pump 2 and Pump 3. Upon zebrafish detection within the field of view, Pump 3 halted while Pump 2 initiated the transfer of zebrafish larva from the loading module to the drug exposure and imaging module. The core of this proposed system was the microfluidic chip with multiple channels in the drug exposure and imaging module (Fig. 2b, Supplementary Fig. 2). Every single larva was loaded into the chip through the Larva inlet. Before and after the transportation of the larva to the Main channel, Pump 4 was actuated to pump out air to form the droplets that encapsulated single-animals, with droplet length matching the distance from the Air inlet to the Liquid outlet. When the droplet passed through the Drug exposure inlet, the AISS controlled Pump 5 to inject drug with specific volume into the droplet to achieve drug exposure with the desired final drug concentration. Then the droplet containing a zebrafish larva and injected compound was transported to the Observation channel 1. If the direction of the larva was detected to be tail-forward, the larva would be immobilized and orientated in the Observation channel 1. Otherwise, the animal (with head-forward direction) would be immediately and carefully pushed into the Observation channel 2 from the Observation channel 1by the AISS. Subsequently, the motorized X-Y-Z stage would be controlled to move the Camera 3 to the corresponding positions for optical evaluation of chemical induced responses of the zebrafish larva. Figure 2c showed the complete automated manipulation process of a single larva within our microfluidic chip in AISS, including animal loading, larva transportation, drug exposure, organ imaging and animal releasing, showing the precise fluidic control of our proposed system.

To further validate the functionality of the AISS for organ-specific evaluation, several well-developed zebrafish transgenic lines (including Elavl3:H2B-GCaMP6f, Kdrl1:eGFP, Apo14: GFP and Cmlc2:eGFP) were employed. Specifically, the special trapping structural design in the observation channel was used to allow orientation and immobilization of the larva in droplet (Supplementary Fig. S2). A similar strategy has been demonstrated in our previous studies^27–29^. After positioning the larva in the Observation channel 1/2 in the microfluidic system, a lateral orientation of the immobilized animal was quickly achieved and thus rendering the capability to microimaging targeted organ systems. Coupled with well-developed zebrafish transgenic lines (e.g. Apo14:GFP, Cmlc2:eGFP, Kdrl1:eGFP, and HuC:eGFP), our system was readily to be utilized in imaging of specific organ, including the liver, heart, vessel and brain (Fig. 2d–g).

### Computer vision-based closed-loop controlling in AISS

**Fig. 3:**
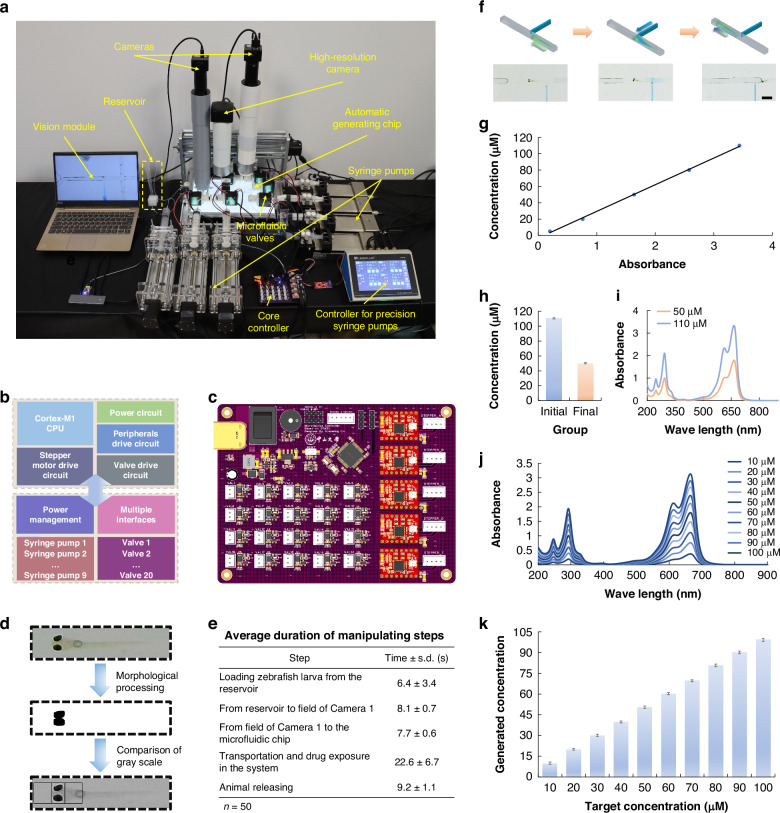
Design and characterization of the precise fluidic control in the AISS. **a** Photograph of the AISS, showing control components. **b**–**c** Block diagram (**b**) and photograph (**c**) showing the key components of the AISS, including microcontroller circuit, power management circuit and motor drive circuit. **d** Image processing in the computer vision program for automatically tracking the coordinates of a zebrafish and orientation determination. **e** Table showing the average duration of the animal manipulating steps in the AISS during an experimental cycle. **f** Schematics and photographs showing the control of drug exposure. Scale bar, 2 mm. **g** Determination of the molar absorptivity of methylene blue based on the detection wavelength at 664 nm; five standard solutions with different concentrations (5 μM, 20 μM, 50 μM, 80 μM, 110 μM) were utilized. **h** Comparison of the concentration (**h**) and absorbance (**i**), between the initial solution and the final solution after 1-minute incubation and transfer in the AISS. **j**, **k** The absorbance (**j**) and quantitative concentration gradients (**k**) of the methylene blue solutions ranging from 10 μM to 100 μM, generated by the AISS. Error bars indicated the standard error of the mean (s.e.m.); *n* = 5

Figure 3a shows the operating system and detailed components of the AISS. Due to the necessity of controlling numerous high-power microfluidic valves and stepper motors, a four-layer printed circuit board (PCB) was designed as the core controller coupling with a computer (Supplementary Fig. 3). This closed-loop controlling system comprised: (1) a microcontroller based on ARM Cortex-M1 architecture (STM32F103RCT6, STMicroelectronics), which was deployed for embedded control programs to enable high-speed computation and rapid response; (2) a power management circuitry for efficiently converting the input 12V to a low-ripple 3.3V via linear voltage regulator circuits to simultaneously powering various components; (3) multiple driving circuits, including stepper motor driving circuits based on a microstepping motor driver (A4988SETTR-T, Allegro) and microfluidic valve driving circuits adopting N-channel Metal-Oxide-Semiconductor Field-Effect Transistor (NMOSFET) low-side driver design (Fig. 3b, c). Importantly, the computer vision-based closed-loop controlling program in AISS relayed on the real-time processing of the images acquired from Camera 1 and Camera 2 was developed (Supplementary Fig. 4). Morphological processing including filtering, binarization, dilation, and erosion, was utilized to enhance the eye’s features of the zebrafish larva, facilitating precise coordinate tracking and determination of the animal’s direction (Fig. 3d). Practically, our system enabled appropriate loading and orientation with a success rate of 95% (*n* = 20). The tests results revealed that a complete manipulation cycle of a behaving zebrafish larva (5 days post fertilization, dpf) in the AISS was less than one minute (Fig. 3e).

### On-chip generation of chemical gradients

Owing to the precise control, the chemical exposure in each droplet was enabled in the AISS. Once Camera 2 detected the passed droplet within the injection range, the initial drug concentrate with specific volume would be injected via Pump 5 (Fig. 3f). To characterize the accuracy of the concentration gradients that generated in the droplets by our system, we employed methylene blue solutions to represent the drug conditions. A concentration-absorbance fitting curve (*R*² = 0.999) was established by detecting five different concentrations of standard methylene blue solutions using a microplate spectrophotometer (Fig. 3g). Subsequently, a 110 µM methylene blue solution was prepared to represent the drug concentrate, which was injected to a droplet by the AISS to the expected 50 µM final concentration to test the accuracy of drug exposure in our system. Figure 3h shows the spectral curves of the two solutions. The final solution had an average concentration of 50.34 µM with a standard error of the mean (s.e.m.) of 0.91, confirming the precision of the drug concentration generation in the AISS. We then utilized the AISS to continuously generate methylene blue solutions of multiple concentration gradients, which were also further confirmed via concentration-absorbance fitting using the microplate spectrophotometer (Fig. 3j, k). These results confirmed that the AISS could be employed to precisely generate chemical gradients in droplets for further in vivo functional profiling.

### Biosafety assessment of the AISS

We next conducted a health assessment of 40 larvae based on both functional and morphological criteria following their processing through our AISS^22,30^. In a 4-day assessment, no significant difference in the cardiac functions, locomotion behaviors or morphological abnormalities was detected between the screened larvae and freely moving animals in the culture dish (Supplementary Fig. 5). Remarkably, even on the fourth day after a 24-h trapping in the AISS, we observed a high survival rate of 95.0 ± 3.1% and a low abnormal rate of 5.0 ± 3.1%. Additionally, all larvae released from the system showed good locomotion behaviors after completing a full experimental cycle in the AISS. The larval behavior and movement were monitored for 15 minutes after a 2-minute light stimulation in the following 4 days. Compared to the freely moving animals, there was no significant difference both in the velocity and traveling distance (Supplementary Fig. 5c, d). Collectively, these results show that our system has enabled the automatic manipulation of living zebrafish larvae quickly and safely, which holds promise for enhancing in vivo screening.

### In vivo cardiac evaluation enabled by the AISS

**Fig. 4:**
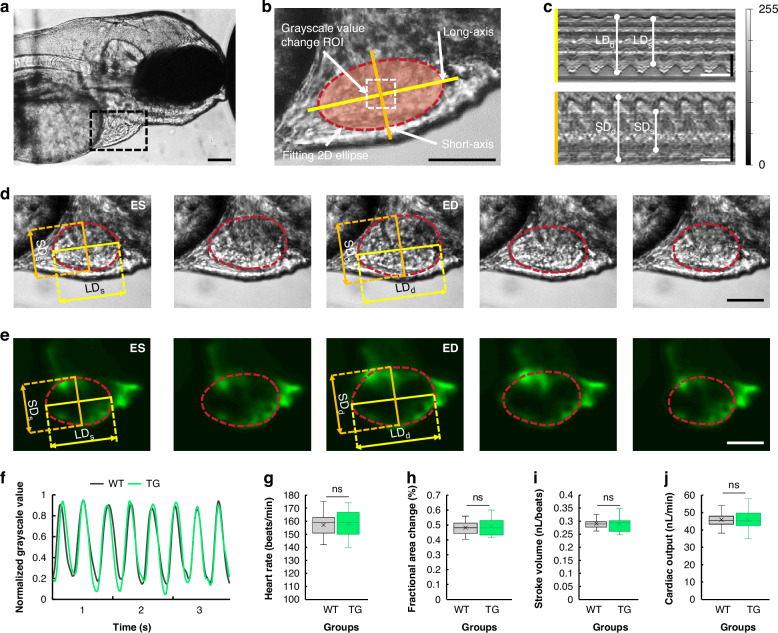
In vivo cardiac evaluation. **a** Images of a larva immobilized in the Observation channel 1/2 with lateral orientation. Scale bar, 100 μm. **b** Zoom-in images of the black rectangle in (**a**) showing the fitting ellipse with a long axis and a short axis, grayscale value changes in the region of interest (ROI) at the ventricular center. Scale bar, 100 μm. **c** Analysis of ventricular wall motion during cardiac cycles by fitting a 2D ellipse to the long and short axes. The maximum and minimum values of the long and short axes were measured as: the long-axis diameter at end-diastole (LD_d_), the long-axis diameter at end-systole (LD_s_), the short-axis diameter at end-diastole (SD_d_) and the short-axis diameter at end-systole (SD_s_). White bar, 500 ms. Black bar, 50 μm. **d**, **e** Sequential images of a cardiac cycle, from end-diastole (ED) to end-systole (ES), in wild-type zebrafish (WT) (**d**) and transgenic zebrafish (TG) (Cmlc2:eGFP) (**e**). Scale bar, 100 μm. **f** The plots of grayscale value changes at the ventricular center for both WT and TG zebrafish. **g**–**j** Quantitative comparisons of heart rate (**g**), fractional area change (**h**), stroke volume (**i**), and cardiac output (**j**) revealed no significant differences between WT and TG zebrafish. Error bars indicated the standard error of the mean (s.e.m.); *n* = 15, “n.s.” stands for no significant difference by one-way analysis of variance (ANOVA)

As shown in Fig. 4a, b, owing to the precise positioning of the animal in the AISS, wild-type zebrafish larva could also be used to enable deep profiling of the organ systems, such as the heart. In this study, a more intuitive analyzing method was employed to describe and capture ventricular motion in the heart. First, the heart rate (HR) could be easily calculated based on the fluctuation of the grayscale values in the center region of the ventricle (Fig. 4b). Then, the ventricular wall motion could be analyzed practically using methods similar to the motion mode (M-mode) echocardiography^31^, enabling continuous tracking of the position changes of ventricular wall throughout the cardiac cycles (Fig. 4c). A two-dimensional ellipse was automatically fitted based on the edge of the ventricle, allowing delineation of the corresponding short-axis and long-axis of the heart using home-developed codes. And the specific lengths of both axes could be measured based on changes of the ventricular edge. Cardiac motion is a cyclic process involving two states: diastole and systole. At the end of diastole and systole, the maximum and minimum values of the long and short axes could be measured as: the long-axis diameter at end-diastole (LD_d_), the long-axis diameter at end-systole (LD_s_), the short-axis diameter at end-diastole (SD_d_) and the short-axis diameter at end-systole (SD_s_), respectively (Fig. 4d). Based on these four parameters, the fractional area change (FAC) of ventricle, which reflected the magnitude of a zebrafish larva's ventricular contractile ability, could be further calculated. Similarly, the stroke volume (SV) and cardiac output (CO) would also be determined to evaluate the animal’s cardiac contractile function. We then compared the HR, FAC, SV and CO between the wild-type zebrafish groups (WT) and transgenic (Cmlc2:eGFP, green fluorescence indicator of cmlc2:EGFP expressed in heart) zebrafish groups (TG) (Fig. 4e). The quantitative results showed that no significant difference between the WT and TG groups was observed in all the assessments (Fig. 4f–i). Therefore, deep profiling of cardiac function in zebrafish using the AISS could be achieved by simply employing wild-type zebrafish and bright-field microimaging, without the need for transgenic zebrafish and fluorescent microscopy.

### Pharmaceutical evaluation using the AISS

**Fig. 5:**
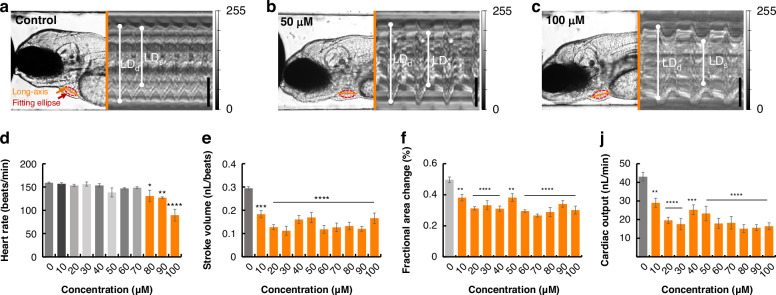
Drug evaluation using the AISS. **a**–**c** Representative images from the control group, the 50 µM sertindole-treatment group, and the 100 µM sertindole-treatment group, showing the changes in ventricular wall motion with increasing drug concentrations. The maximum and minimum values of the long-axis were measured as: the long-axis diameter at end-diastole (LD_d_) and the long-axis diameter at end-systole (LD_s_). Scale bar, 50 μm. **d**–**j** Quantitative analysis of cardiac toxicity, measured by heart rate (**d**), fractional area change (**e**), stroke volume (**f**), and cardiac output (**j**), with treatments of 10 different concentrations of sertindole ranging from 0 µM to 100 µM. Error bars indicated the standard error of the mean (s.e.m.), *n* = 5. *p < 0.05, **p < 0.01, ***p < 0.001, and ****p < 0.0001 by one-way analysis of variance (ANOVA)

As we demonstrated above, our AISS system enabled rapid manipulation of oriented-zebrafish in droplets and precise liquid control, facilitating specific organ imaging and chemical gradients exposure. Hence, to validate the capability of the AISS for quick pharmaceutical evaluation, we examined the cardiac sensitivity to the gradients of an atypical antipsychotic, sertindole^32^. Sertindole was initially used to treat psychiatric disorders but was later reported to have cardiotoxic effects, making it an ideal drug for assessing cardiac toxicity in our proposed system. Actually, the drug treatment duration was allowed to be tailored to meet different testing requirements in our system. As shown in Supplementary Fig. 6, drug treatment duration could be precisely tailored from 0 to 60 minutes, and the cardiotoxicity of 50 μM sertindole in zebrafish larvae was clearly observable, with the onset occurring at 10 minutes. Therefore, the 10-minute exposure was used to quickly screen the cardiotoxicity of the drug with different concentrations. 10 concentration gradients of sertindole, ranging from 10 to 100 µM, were generated and implemented in the cardiac toxicity screening in the AISS (Fig. 5). The representative images from the control group, the 50 µM sertindole treatment group, and the 100 µM sertindole treatment group, showed that increasing concentration significantly decreased the heart rate (Fig. 5a–c). According to the grayscale change curves, ventricular movement gradually became obstructed, with lower smoothness compared to the control group.

To further profile cardiac toxic effects of sertindole in vivo, the chemical induced changes of the HR, FAC, SV and CO of the animals were then evaluated using our AISS. As shown in Fig. 5d, the HR decreased significantly during exposure of sertindole with higher concentrations. Compared to 159 beats/min in control groups, the average HR was slowed down to 89.6 beats/min in 100 µM sertindole treated groups. Next, the SV, which refers to the volume of blood pumped out of the ventricle during each heartbeat, was analyzed with all the gradients (Fig. 5e). After sertindole treatment, the average SV decreased significantly by approximately 0.15 nL compared to the control groups. Moreover, the FAC significantly decreased even during the exposure of sertindole with low concentrations, indicating weakened ventricular contractility after sertindole treatment. And the CO gradually decreased, from 43 nL/min in the control group to approximately 16.47 nL/min after treatment with 100 µM sertindole, demonstrating a significant reduction in blood circulation efficiency after sertindole treatment. Overall, it showed that sertindole with low concentration would induced cardiac toxicity effects although the heart rate only decreased under the exposure of high-concentration sertindole. This suggested that solely relying on HR, the commonly used cardiac function indicator, might not be sufficient for the quick detection of cardiotoxicity during early stages of acute testing, particularly at lower concentrations. Furthermore, it emphasized the necessity of incorporating multiple indicators for quick cardiotoxicity evaluation. Collectively, these indicators allowed rapid identification of potential cardiotoxicity, eliminating the need for prolonged observation with wider concentration ranges, shortening the screening period and achieve a less compound consumption. These results suggested successful functional, multiple-dimensional profiling of compounds in specific organs of zebrafish, further exemplifying the power of the proposed fully-automated system for in vivo screening.

## Conclusion

Recent studies have demonstrated the functionality of microfluidic systems for organ investigation and drug screening using zebrafish^26–28,33–35^. However, these systems involved complicated operations for zebrafish larvae transportation and immobilization, requiring manual intervention to varying degrees, and struggle to meet the needs of low drug consumption. In this study, we developed and validated an automated in vivo screening system (AISS), which integrates microfluidic manipulation technique with fully automated computer-vision feedback control methods for drug evaluation. By virtue of precise fluidic control, this system enabled on-chip encapsulation of single zebrafish larva into a droplet and acute drug exposure with multiple concentration gradients. Importantly, each droplet containing single-larva had a fluidic volume as low as 5.56 microliters, significantly reducing the amount of drug consumption. The AISS largely simplified animal manipulation, drug treatment, and optical microimaging of specific organs inaccessible in previous systems. However, the AISS could only handle one larva at a time, which limited the throughput of our system. Even though the exposure time could be tailored to achieve a higher efficiency, the AISS presented in this study was ready to be further refined in several ways. For example, arraying design of our microfluidic system could be applied to enable simultaneous process of multiple larvae^36,37^; more advanced droplet-based techniques could also be engaged in our system to allow on-chip assessing compounds in large-scale^38,39^. Overall, we believe that the AISS holds significant potential for cost-effective and time-efficient animal manipulations, transportation, and evaluations in zebrafish studies, offering a new paradigm for automated in vivo functional screening.

## Materials and Methods

### Zebrafish line

The wild-type zebrafish and the transgenic zebrafish line Elavl3:H2B-GCaMP6f, Kdrl1:eGFP, Apo14: GFP and Cmlc2:eGFP were maintained in aquaria under standard laboratory conditions (at 28 °C under a cycle of 14 h light, 10 h dark). Larvae of 5–7 dpf were used in the drug screening experiments. All animal work was carried out with prior approval from the animal ethical committee of Sun Yat-sen University and was in accordance with local animal care guidelines.

### Fabrication of the microfluidic chip

Supplementary Fig. 7 illustrates the fabrication process of the microfluidic chip used in the AISS. The molds of microfluidic chip were machined using a high-precision computer-numerical-controlled machine (CNC, Dingsheng CNC Equipment Co., Ltd.) with a copper plate. After machining, the molds were soaked in 100 % alcohol overnight and subsequently rinsed multiple times. Polydimethylsiloxane (PDMS) was then cast onto the prepared convex molds. Following the evacuation of air bubbles in a vacuum chamber, the PDMS-coated mold was cured at 80°C for 5-6 hours. The cured PDMS layer was then demolded and further processed to create the inlets and outlets. Finally, a plasma treatment (Shenzhen Sanhe Boda Electromechanical Technology Co., Ltd.) was employed to permanently bond the microfluidic layer to the glass substrate, resulting in the final microfluidic chips utilized in this study.

### Fabrication of PCB

One of the key components of our system is the control board, which is assembled from two PCBs (Supplementary Fig. S4a, b). To reduce the volume and improve current carrying capacity, both the core board and the motor drive module were designed with four layers of copper foil. We used glass fiber cloth as a reinforcing material, bonded with epoxy resin and a copper-clad plate (FR-4, JIALICHUANG) to fabricate the PCB. Lead-free low-temperature soldering paste (LF999; KELLY SHUN) was employed to attach various surface-mounted components, including the ARM Cortex-M1 MCU (STM32F103RCT6; STMicroelectronics), DC-DC converter (TPS54302DDCR, Texas Instruments), microstepping motor driver (A4988SETTRT, Allegro), Low Drop Regulator (AMS1117, Puolop), N-Channel MOSFETs (AO3400A, Born), along with various surface-mount resistors, capacitors, and diodes by heating at 230 °C (Supplementary Fig. S4c–f).

### Software development

The software development consisted of two primary parts: (i) the embedded system tailored for the STM32F103RCT6 SoC and (ii) the OpenCV-based computer-vision program.

The firmware of the STM32F103RCT6 was written in C and utilized the standard peripherals library. The firmware enabled the core board to receive serial port commands from computer vision program and control individual electrical modules with rapid responsiveness. Specifically, it facilitated the execution of various pre-packaged operating modes, including flexible control of microfluidic valves and stepper motors to achieve high-precision fluidic control.

The computer-vision program was developed in Python, which was capable of issuing control commands to the core board and the motorized X-Y-Z stage through a serial port while processing visual tasks, enabling the system to achieve closed-loop control based on visual feedback. Its design consisted of two parts. First, the visual detection in the loading module: After simple filtering and binarization processing of the images acquired from Camera1, the presence of zebrafish in the field of view of Camera1 could be efficiently determined, enabling the system to seize the appropriate moment to transfer the zebrafish larva into the drug exposure and imaging module. Second, the visual detection in the drug exposure and imaging module: After performing median filtering, binarization, erosion, dilation, and other morphological processing on the images acquired by Camera2, important features such as the eyes of the animals could be accurately segmented and used to enable coordinate tracking and corresponding control commands sending (Supplementary Fig. S3).

### Zebrafish reservoir

The zebrafish reservoir was a cone-like container with a base radius of 8 mm, connected to silicone tubes at both the top and bottom. Air was injected into the reservoir at a rate of approximately 45 μL/s for generating air bubbles to randomly lift the zebrafish larva. By employing the pumps, the top tube generated a negative pressure at a rate of ~35 μL/s to draw the zebrafish.

### Concentration validation

An ultraviolet-visible micro-spectrophotometer (K5800C, Beijing Kaiao Technology Development Co., Ltd.) was used to validate the final concentrations of the solutions of methylene blue based on the proportional relationship between the ultraviolet absorbance and solution concentration. Samples with five concentrations of 5 µM, 20 µM, 50 µM, 80 µM, and 110 µM were prepared for the determinations of the molar absorptivity of the methylene blue. This molar absorptivity curve was then used to determine the final concentration of the diffused solutions in the droplets in this study.

### Imaging

In the AISS, three cameras were utilized. Two of these cameras (MT9P031, Onsemi) were employed for visual control, operating at 30 fps during visual processing. The third camera, a high-resolution camera (IMX183, Sony) was utilized for data acquisition, achieving a frame rate of 35 fps. For fluorescent imaging, a fully automated inverted fluorescent microscope (Leica THUNDER DMi8) equipped with a cooled high-speed CMOS camera (DFC 9000GT) and a 10× (NA, 0.4) objective. LAS X software was installed to control the microscope. When performing microscopic real-time imaging on the larva to obtain data, the camera was operated at 10 Hz frame rate to ensure that no information was missed.

### Chemical treatment

In this study, 10-min treatment of sertindole was performed, followed by a 1-minute data collection period. Sertindole was dissolved in DMSO as a vehicle to create 10 mM stock solutions. Sertindole was purchased from Topscience Co. Ltd.

### Assessment of cardiac function

The indicators of the cardiac functions were calculated based on the grayscale value changes around the center of the ventricle. Specifically, the raw data was firstly extracted and filtered by a Butterworth bandpass filter with frequency of 0.2~2Hz to remove the noise. Then the filtered data would be normalized for further analysis of the cardiac function including the heart rate (HR), the fractional area change (FAC), the stroke volume (SV) and the cardiac output (CO).

First, the peaks of the normalizing data were further automatically counted to obtain the HR value.

By fitting a two-dimensional ellipse to the ventricle's edges, corresponding long and short axes could be delineated. This enabled the tracking of continuous changes in the ventricular wall's position throughout the cardiac cycle by identifying a linear region of interest (ROI). We developed codes to automatically measured the intensity of pixel grayscale values along the axes at each point, recording these values in a two-dimensional matrix (*I*).

$$I\equiv \left[\begin{array}{cccc}{l}_{0,0} & {l}_{1,0} & \ldots & {l}_{t,0}\\ {l}_{0,1} & {l}_{1,1} & \ldots & {l}_{t,1}\\ \vdots & \vdots & \vdots & \vdots \\ {l}_{0,m} & {l}_{1,x} & \ldots & {l}_{t,x}\end{array}\right]$$

In this matrix, the first coordinate (*t*) represented the sequential image number in the series, while the second coordinate (*x*) represented the position of pixels along the ROI line. Thus, any specific value *l*_*t,x*_ in the matrix represented the intensity (an integer value between 0 and 255) at position *x* along the line in frame *t*. Consequently, the specific lengths of the long axes (*D*_*L*_) and short axes (*D*_*S*_) were measured based on the changes in the ventricular edge, and the ventricular area was approximated through the calculation:

$${\rm{Area}}=\frac{1}{4}\times \pi \times {D}_{L}\times {D}_{S}$$

Subsequently, at end-diastole (ED) and end-systole (ES), the maximum (EDA) and minimum (ESA) values of ventricular area could be respectively calculated, thus allowing the calculation of the fractional area change (FAC) of the ventricle:

$${\rm{FAC}}=100\times \frac{({\rm{EDA}}-{\rm{ESA}})}{{\rm{EDA}}}$$

The FAC could be used to indicate the magnitude of the zebrafish ventricular contractility, with a larger FAC representing stronger contractility. Similarly, by calculating the volume of the fitted ellipse based on its *D*_*L*_ and *D*_*S*_, we can determine the ventricular volume:

$${\rm{Volume}}=\frac{1}{6}\times \pi \times {{\rm{D}}}_{{\rm{L}}}\times {{\rm{D}}}_{{\rm{S}}}^{2}$$

By subtracting the ventricular volume corresponding to ED from that of ES, the stroke volume (SV), which was used to represent the amount of blood ejected by the ventricle during heart beating, was then obtained:

$${\rm{SV}}=({\rm{EDV}}-{\rm{ESV}})$$

Where end-diastolic volume (EDV) was the volume of the ventricle when it was filled during diastole; and end-systolic volume (ESV) represented the volume of the ventricle at the end of systole.

Combining SV and HR, the Cardiac output (CO) could be calculated:

$${\rm{CO}}\,({\rm{nanoliter}}/\!\min)={\rm{SV}}\,({\rm{nanoliter}}/{\rm{beat}})\times {\rm{HR}}\,({\rm{beats}}/\!\min )$$

### Statistical analysis

The bar graph data were presented as the mean ± standard error of mean (s.e.m.). For the box plots, the vertical centerline indicated the median, while the width of the box and error bar represented the interquartile range (IQR) and 1.5 times the IQR, respectively. An independent samples t-test was used to calculate differences between the two groups. Statistical analysis was performed by one-way analysis of variance (ANOVA) using SPSS Statistics 25 (IBM Corp., Armonk, NY). The difference between groups was considered statistically significant for *p < 0.05, **p < 0.01, ***p < 0.001, and ****p < 0.0001.

## Supplementary information

Supplementary Movie S1. The view of camera 1 in loading module during working

Supplementary Movie S2. The view of camera 2 in the drug exposure and imaging module during working

Supplementary Movie S3. Heart activities recordings using the AISS

Supplementary Materials Clean Version

## Data Availability

The primary data that support the findings of this study are available from the corresponding author upon request.
