# Supplementary material for: Fully automated in vivo screening system for multi-organ imaging and pharmaceutical evaluation
Source: Microsyst Nanoeng. 2025 Jan 27;11:22. doi: 10.1038/s41378-024-00852-9 (PMC11770100; doi:10.1038/s41378-024-00852-9)

**Fully Automated *In Vivo* Screening System for Multi-organ Imaging and Pharmaceutical Evaluation**

Junhan Duan^1,2^, Guanming Lin^1,2^, Kangjian Jiao^1^, Xiaohui Hong^1^, Xudong Lin^1*^

^1^Guangdong Provincial Key Laboratory of Sensor Technology and Biomedical Instrument

School of Biomedical Engineering

Shenzhen Campus of Sun Yat-Sen University

Shenzhen, 518000, China

^2^These authors contributed equally to this work

*Correspondence should be addressed to

Dr. Xudong Lin, [linxd37@mail.sysu.edu.cn](mailto:linxd37@mail.sysu.edu.cn)

**Supplementary Figures**


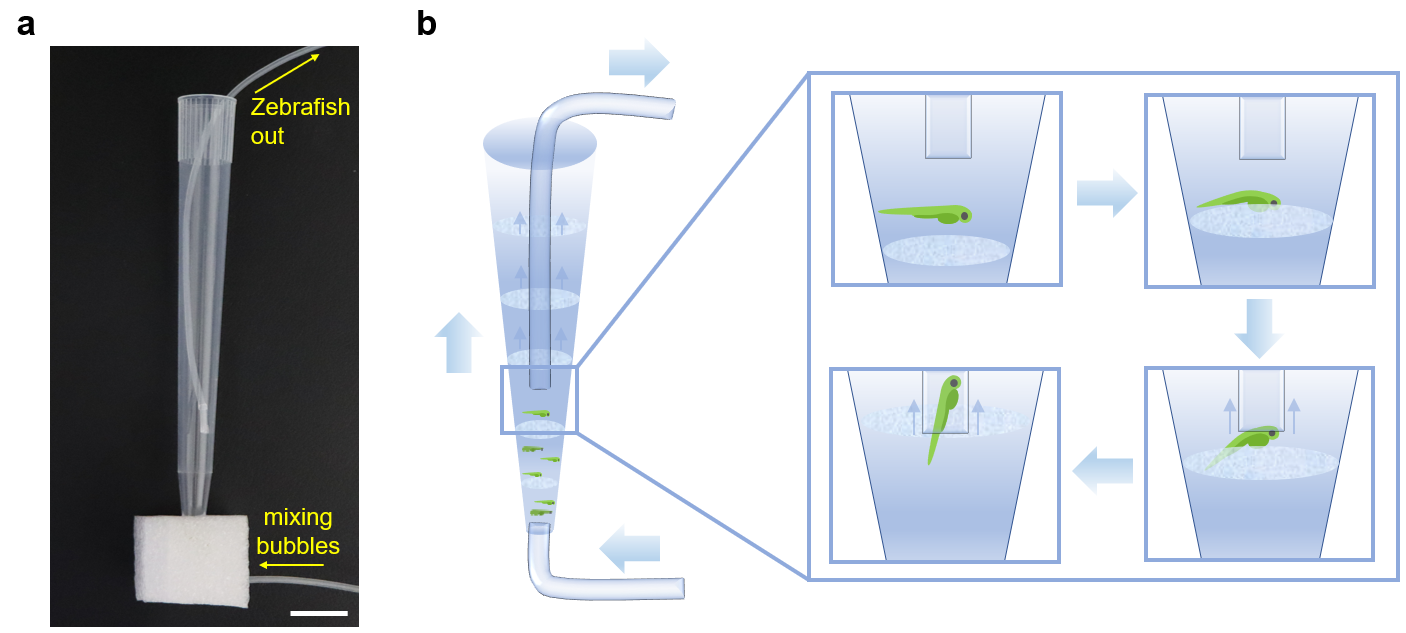


**Supplementary Figure S1**. **The tapered reservoir capable of generating air bubbles for automatic loading of zebrafish larvae. a**, Images of the loading module consisted of an air bubble generator, tapered reservoir, and a negative-pressure collection silicone tubing. Scale bar, 2 cm. **b**, Schematic showing the air bubbles aided automated loading of zebrafish larvae. Coupling with a negative-pressure silicone tubing, bubbles were generated from the bottom, facilitating the intake of zebrafish larvae from above.


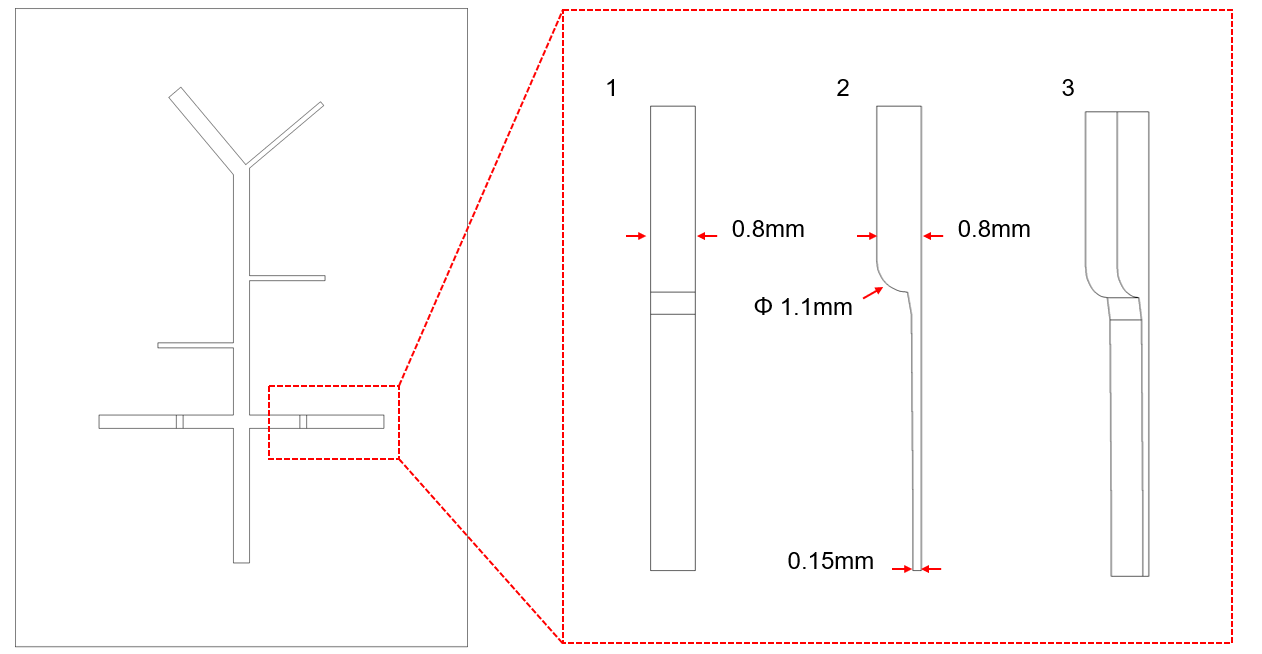


**Supplementary Figure S2**. **Detailed designs of the microfluidic chip.** Insets illustrations show the top view (1), side view (2) and 3D view (3) of the chip respectively.


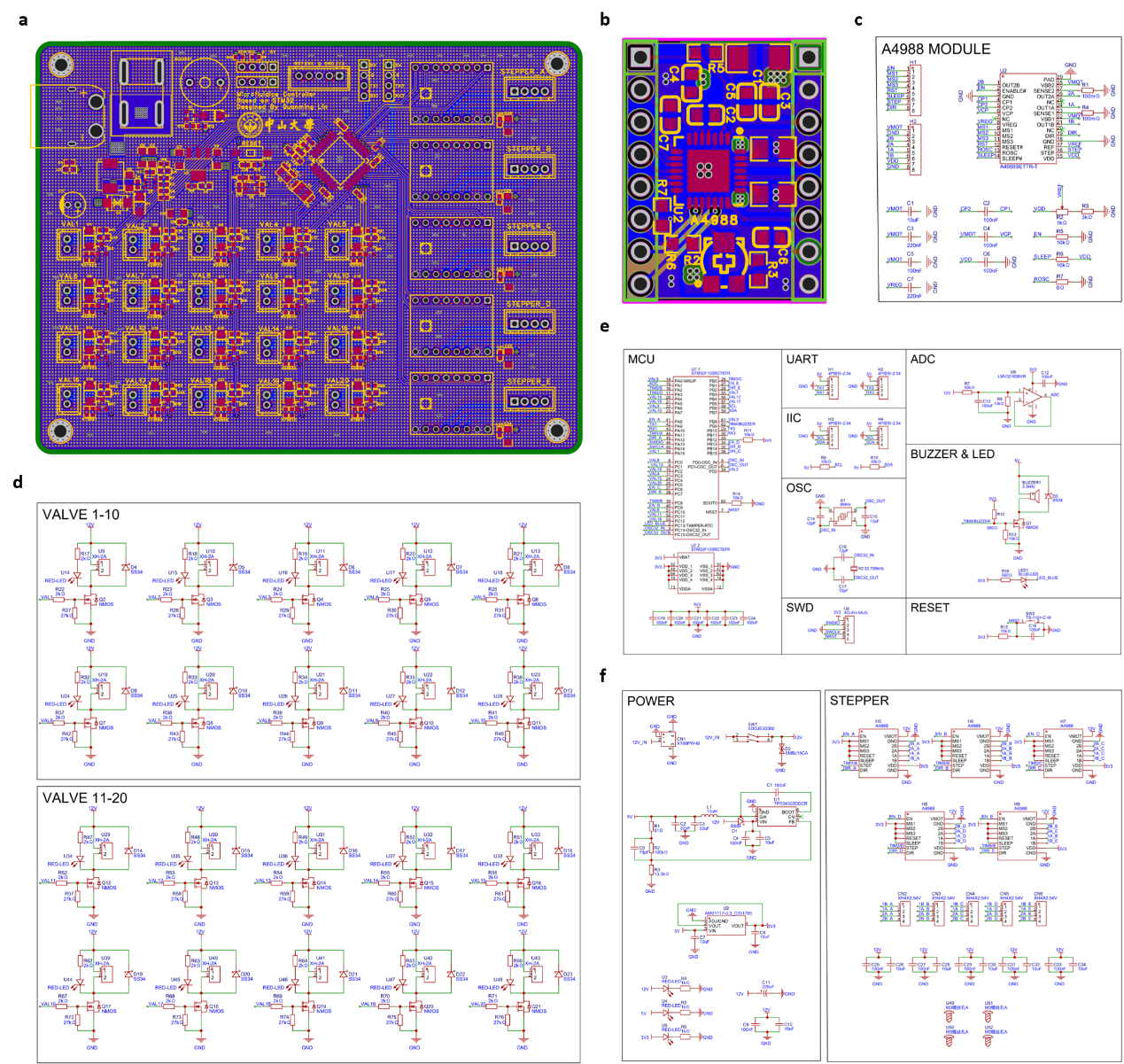


**Supplementary Figure S3**. **The PCBs design. a-b**, PCBs design containing component layout and wiring conditions. **c-f**, Schematic diagram showing all of the electrical circuits in our design.


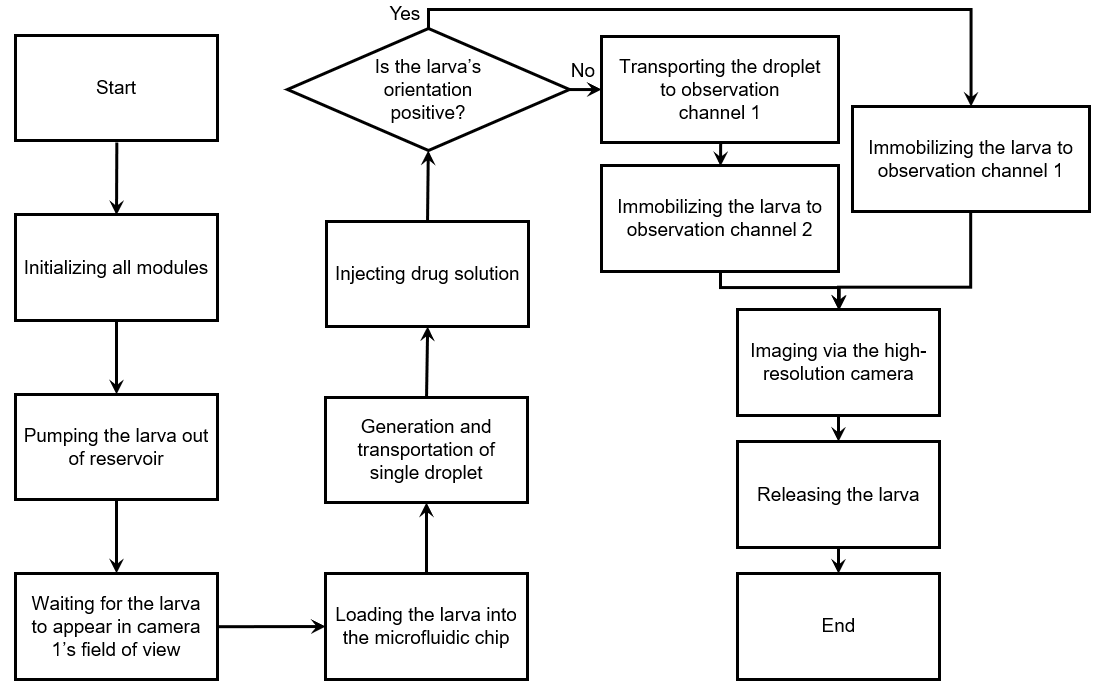


**Supplementary Figure S4. The flowchart of the main program.**


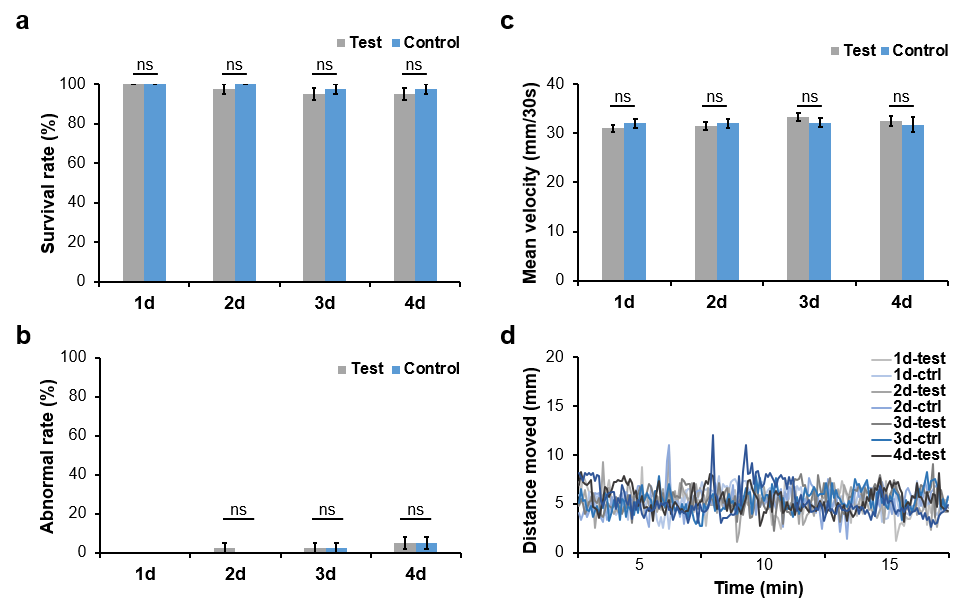
**Supplementary Figure S5**. **Quantitative health assessment of the larvae released from the AISS.** **a-b**, Survival rate (**a**) and abnormal rate (**b**) of zebrafish larvae in the following 4 days after 24-hour trapping in the AISS. **c-d,** Behavioral data analysis of zebrafish larvae including the mean velocity (**c**) and the distance moved (**d**) in the following 4 days after 24-hour trapping in the AISS. Average movement distance comparison between the test group and the control group every day, and the representative movement results of each day. A total of 40 larvae were evaluated from five independent experiments (8 larvae/experiment). Error bars indicated standard error of mean (s.e.m); “ns” indicated no significant difference by one-way analysis of variance (ANOVA).


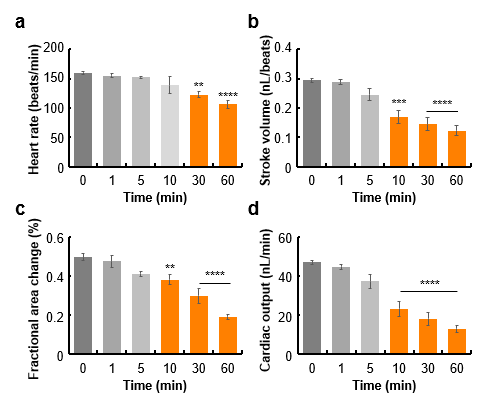


**Supplementary Figure S6**. Cardiotoxicity induced by 50 μM sertindole with various treatment durations. Quantitative analysis of cardiac toxicity, measured by heart rate (**d**), fractional area change (**e**), stroke volume (**f**), and cardiac output (**j**). Error bars indicated the standard error of the mean (s.e.m.), n = 5. *p < 0.05, **p < 0.01, ***p < 0.001, and ****p < 0.0001 by one-way analysis of variance (ANOVA).


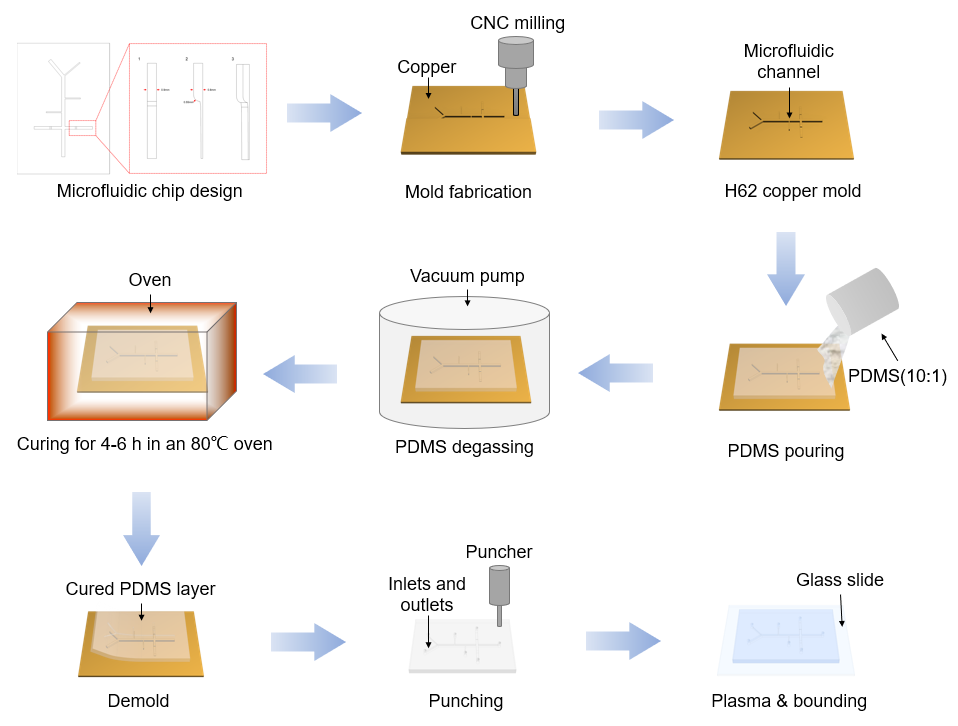


**Supplementary Figure S7**. **Fabrication of microfluidic chip based on computer numerical control (CNC) machining.**

**Supplementary Movies**

**Supplementary Movie S1**. The view of camera 1 in loading module during working.


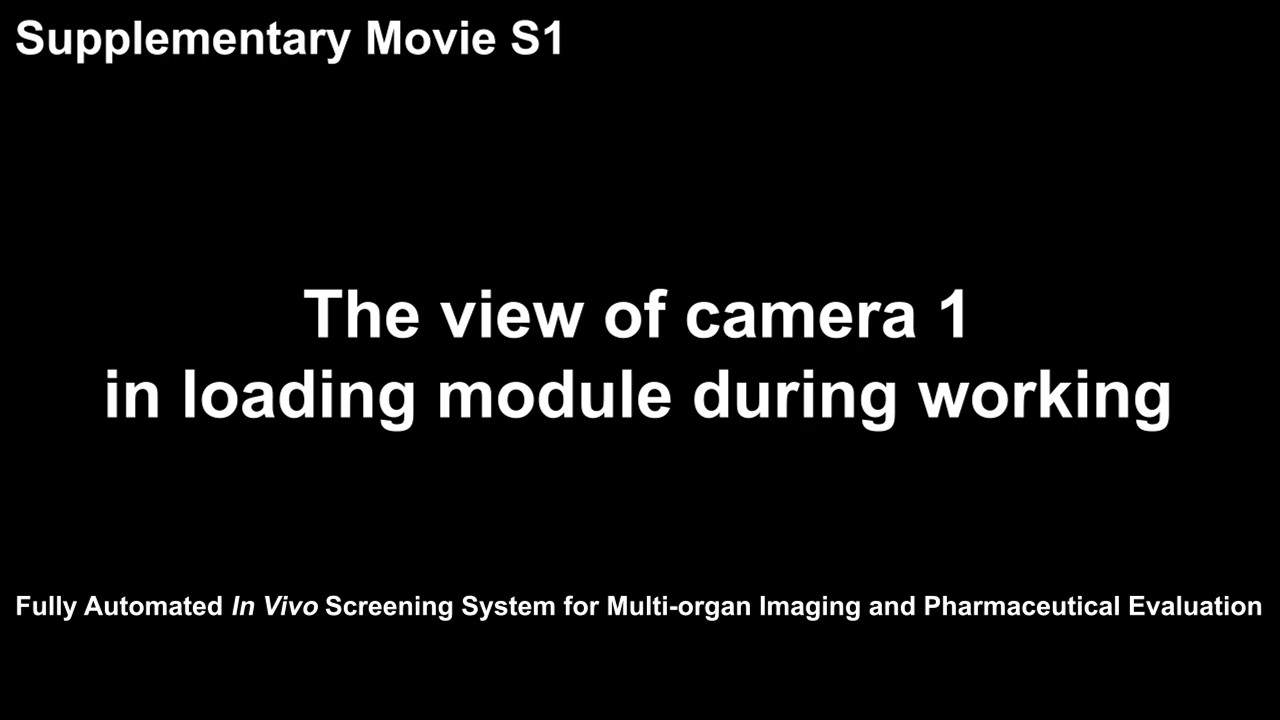


**Supplementary Movie S2**. The view of camera 2 in the drug exposure and imaging module during working.


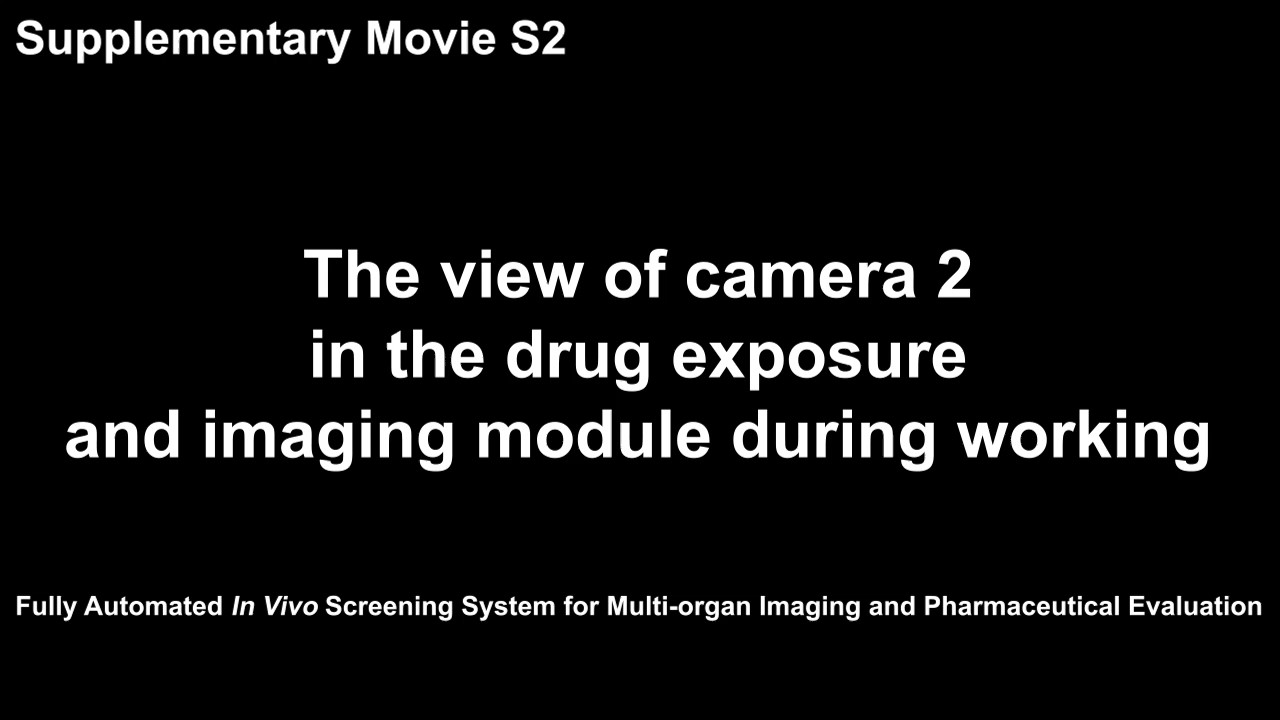


**Supplementary Movie S3**. Heart activities recordings using the AISS.


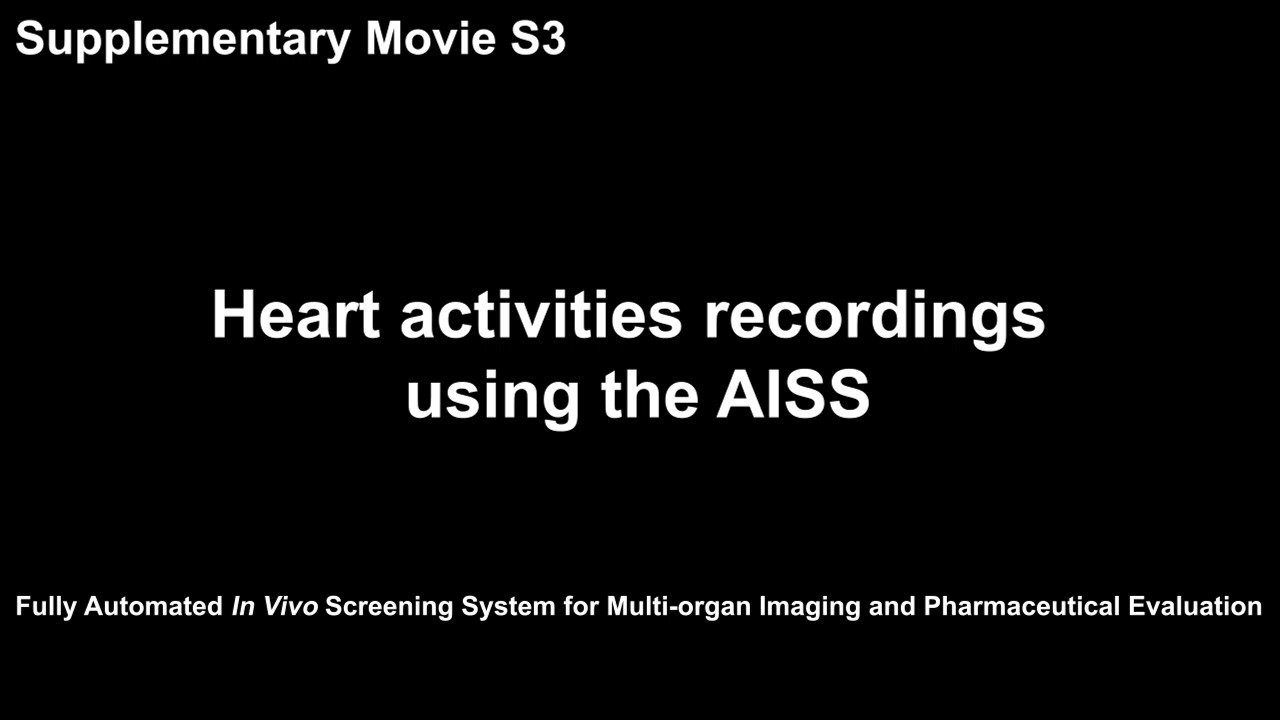

Supplement: Supplementary file 4 — Supplementary Materials Clean Version [file 41378_2024_852_MOESM4_ESM.docx]
